# Identification of low penetrance alleles for lung cancer: The GEnetic Lung CAncer Predisposition Study (GELCAPS)

**DOI:** 10.1186/1471-2407-8-244

**Published:** 2008-08-20

**Authors:** Tim Eisen, Athena Matakidou, Richard Houlston, the GELCAPS Consortium

**Affiliations:** 1 Department of Medical Oncology, University of Cambridge, Cambridge CB2 2RE, UK; 2 Section of Cancer Genetics, Institute of Cancer Research, Sutton, Surrey, SM2 5NG, UK; 3 List of collaborators available on request

## Abstract

**Background:**

Part of the inherited risk to lung cancer is likely to include common, low risk alleles. The identification of this class of susceptibility is contingent on association-based analyses. We established GEnetic Lung CAncer Predisposition Study (GELCAPS) to collect DNA and clinico-pathological data from a large series of cases and a series of spouse/partner controls, thereby generating a key resource for the identification of low risk alleles.

**Methods:**

GELCAPS was one of the first genetic epidemiological trials in the UK to be adopted by the National Cancer Research Network (NCRN) onto its portfolio with the participation of over 100 oncology departments specialising in the management of lung cancer.

**Results:**

Samples from over 5,000 independent lung cancer cases and 2,000 controls have so far been assembled through GELCAPS.

**Conclusion:**

GELCAPS represents one of the largest datasets of its type in the world capable of informing on the contribution of low penetrance alleles to the development of lung cancer and the influence of genetic variation on outcome. In addition our experience in developing the GELCAPS serves to illustrate how large DNA biobanks for genetic analyses can be rapidly generated within the UK using the NCRN.

## Background

Lung cancer is a major cause of cancer mortality worldwide [1]. In the United Kingdom, it accounts for more than 33,000 cancer deaths each year (Cancer Research UK). The disease is frequently cited as a malignancy solely attributable to environmental exposure, principally tobacco smoking. It has, however, long been postulated that individuals may differ in their susceptibility and there is strong evidence from epidemiological studies for a familial risk [reviewed in [2]]. Direct evidence for a genetic predisposition is provided by the increased risk of lung cancer associated with a number of rare Mendelian cancer syndromes, such as in carriers of germline *TP53 *[3]and *RB *[4,5] mutations, as well as in patients with Bloom's [6] and Werner's [7] syndromes.

The two major types of lung cancer, non-small cell lung cancer (NSCLC), and small cell lung cancer (SCLC) account for 75% and 25% of cases respectively. Although the histological features are different between these (reflected in differences in patterns of gene expression), there are similarities in the spectrum of underlying somatic genetic alterations suggesting commonality in pathogenesis. Moreover, the observation that the familial risks are not subtype dependent [8-13] and that histological concordance between affected family members is poor [9] is consistent with the hypothesis of a "generic" inherited susceptibility to lung cancer.

The genetic basis of inherited susceptibility to lung cancer outside the context of the rare Mendelian cancer predisposition syndromes is at present undefined, but a model in which major gene loci account for the excess familial risk seems unlikely. One hypothesis about the allelic architecture of susceptibility proposes that part of the genetic risk is caused by disease loci, which include common, low penetrance alleles. This "common-disease common-variant" hypothesis implies that conducting association analyses based on scans of Single Nucleotide Polymorphisms (SNPs) should be a powerful strategy for identifying low-penetrance variants [14,15].

Previous studies aimed at identifying low penetrance alleles for lung cancer susceptibility have largely been based on a candidate gene approach formulated on preconceptions as to the role of specific genes in the development of the disease. Perhaps not surprisingly most studies have to date only evaluated a restricted number of polymorphisms, primarily in genes implicated in the metabolism of tobacco-associated carcinogens and the protection of DNA from carcinogen-induced damage. However, without a clear understanding of the biology of lung cancer predisposition the definition of suitable genes for the disease is inherently problematic making an unbiased approach to loci selection highly desirable.

Despite much research, few definitive low penetrance susceptibility alleles for lung cancer have been to date unequivocally been identified through candidate-based association studies. As with many other diseases, positive associations have been reported for various polymorphisms of genes such as *GSTT1 *[16], *GSTM1 *[17], *ERCC2 *[18], *CYP1A1 *[19], and *TP53 *[20] from small studies, but few of the initial positive results have been replicated in subsequent studies. The inherent statistical uncertainty of case-control studies involving just a few hundred cases and controls seriously limits the power of such studies to reliably identify genetic variants conferring modest but potentially important risks.

In addition to genetic variation affecting the risk of developing cancer it is increasingly being recognised that genetic variation, not necessarily in the same genes, may also affect clinical outcome. As with case-control association studies aiming to identify novel susceptibility alleles the same issues of study power pertain to the search for prognostic markers and such studies are again contingent on access to large case-series.

Following the sequencing of the human genome, large-scale harvests of SNPs have been conducted and > 10 million documented. Patterns of linkage disequilibrium (LD) between SNPs have been characterised allowing subsets of SNPs (tagging SNPs) to be selected that capture a large proportion of the common sequence variation in the human genome. This coupled with the advent of highly efficient analytical platforms allow whole genome-wide studies (GWAS) for disease associations to be conducted cost effectively. The relationship between patients' genotype and risk of lung cancer is now open for exploration.

The identification of genes associated with cancer predisposition and determination of their contribution to disease incidence are contingent on having DNA samples from large, systematic series of cancer patients. The resulting genetic epidemiological data provides the information on which to base the identification, counselling and management of at-risk individuals. The National Cancer Research Network (NCRN) was established to provide support for clinical cancer research in England and is one of the most substantial and constructive developments in the area of cancer research to be made in recent years in the United Kingdom. In England, serving a population of 50 million people, the NCRN is made up of 34 geographically distinct Networks covering the entire country. Within each Network there are clinical research support staff and infrastructure to promote accrual of patients into trials and studies, and the collection of high quality clinico-pathological data and appropriate biological samples. Hence the NCRN presents a major scientific initiative not only in the field of clinical trials but also in the field of genetic epidemiology.

To create a resource for identifying low penetrance alleles for lung cancer we established GELCAPS (GEnetic Lung CAncer Predisposition Study) in March 1999 to collect DNA and clinico-pathological data from a large series of lung cancer cases. Within 5-years of setting up the initiative by linkage with the NCRN it has been possible to create a world-class resource of biological and clinico-pathological data from over 5,000 individuals with lung cancer.

## Methods/Design

### Eligibility criteria

All patients diagnosed with lung cancer between March 1999 and July 2004 were eligible for the study. To ensure that data and samples were collected from *bona fide *lung cancer cases and avoid issues of bias from survivorship only incident cases with histologically or cytologically (only if not adenocarcinoma) confirmed primary disease were ascertained. Partners of recruited lung cancer patients with no personal history of cancer were recruited as controls.

### Procedural outline

A standardised questionnaire was used to collect basic demographic characteristics-sex, date of birth, ethnic group (White, Black-Caribbean, Black-African, Black-other, Indian, Pakistani, Chinese, Other), country of birth, current area of residence – in addition to details on active and past smoking history (including type of tobacco product, amount smoked, age at first cigarette and age at any major change of smoking habits), exposure to asbestos, occupational history, and personal past medical history. All questionnaires were self-administered and no surrogate responders were used. An open question was used to obtain information on family history of cancer involving first-degree relatives. A positive history of lung cancer was only assigned when detailed information was provided identifying the family member affected by lung cancer. The referring clinician using a standard registration form supplied clinico-pathological details (type of lung cancer, stage at presentation) of patients.

Coupled with patient recruitment their spouses/partners who had no known past or current history of malignancy were invited to participate for the purposes of contributing to the generation of a control series. For these individuals details of sex, date of birth, ethnic group, place of birth, current area of residence and smoking history were collected through a self-administered questionnaire. 10–20 ml EDTA-venous blood samples were collected from all participants. Consent forms, questionnaires, registration forms and blood samples were returned to the Institute of Cancer Research (ICR) by mail. Blood samples collected were stored at -80°C prior to DNA extraction and quantification.

It is our intention to collect outcome data on all cases entered into GELCAPS. In the first stage of this process subsets of participating centers were asked to provide the clinical details on the outcome of the recruited lung cancer patients. Records were requested based on their date of accrual, with those accrued at the beginning of the study being requested first. A standard proforma was used to collect information on diagnosis, stage, treatment and survival. Fully informed consent was obtained from all patients alive at the time of outcome data collection. Outcome forms were returned to the ICR by mail and details were stored electronically.

### Statistical considerations

The primary aim of establishing GELCAPS was to generate a DNA resource of lung cancer patients sufficiently large to robustly identify low penetrance alleles by association studies of genetic polymorphisms. From the outset we envisaged that at some juncture such searches would be conducted on a genome-wide basis. It is well recognised that as such studies involve typing a vast number of markers, a large number of false positive associations will inevitably be generated and only a small number of markers will be truly associated with disease susceptibility. Hence associations need to attain a high level of statistical significance to be established beyond reasonable doubt and significance levels of ~10^-7 ^have been proposed as being appropriate [14]. The original target of GELCAPS was to assemble a series to include ~2,000 cases. This figure had been arrived upon on the basis of upon contemporaneous views of the probable impact of common alleles on disease risk. During development of GELCAPS studies of other common diseases indicated that common disease alleles are likely to be associated with risks typically in the range of 1.1–1.5. To identify alleles conferring such risks is contingent on sample sets twice that of our original target and we therefore revised our target accordingly in order to have ~80% power to identify an association between SNP genotype and risk.

### Ethical considerations

In generating DNA registries such as GELCAPS ethical considerations are central to study design. One of the particular strengths of studies such as GELCAPS is that once constructed the DNA database can be probed repeatedly for different existing and newly identified candidate risk factor genes. It is not feasible to contact all study entrants to seek further written consent for specific test therefore, the information sheet and study discussion was centred on the general concept of 'genetic analyses'. As these investigations were to be solely for research to find new gene(s) predisposing to cancer it was implicit that no individual results will be conveyed to persons. In publications of findings no study entrant would be identifiable. As with all studies of this nature we clearly stated that if a study entrant wished to withdraw their DNA sample and all information held on them would be destroyed. To ensure confidentiality data is held under secure conditions at the ICR Institute of Cancer Research and information held on study entrants will not be divulged to any person or agency without the prior written agreement of the study entrant.

All clinical information and biological samples were obtained only after fully informed consent was obtained from participating individuals, and in accordance with the tenets of the Declaration of Helsinki. Ethical approval for the study was obtained from the London Multi-Centre Research Ethics Committee (MREC/98/2/67) and local ethical committees. Personal information was stored in accordance with the Data Protection Act (1998).

### Extraction of DNA, storage and quality assurance

DNA was extracted from EDTA blood samples using either a standard salt extraction procedure or using the Chemagen system (Chemagen Biopolymer-Technologie AG, Arnold-Somerrfield-Ring 2, 5499 Baeswelder, Germany, Picogreen quantified (Quant-it, Invitrogen, Paisely, UK) and normalised to 100 ug/ul in TE buffer. DNAs stocks are being stored in Eppendorf tubes (Barkhausenwe 1 22339 Hamburg, Germany) at -80°C. To avoid subjecting stock DNAs being to repeated thawing and freezing we have generated a series of "master" 96 deep well plates of samples from which DNAs can be readily robotically abstracted for genotyping studies. Fidelity of DNA is being constantly evaluated by monitoring performance in the different genotyping platforms.

## Results

**Figure 1:**
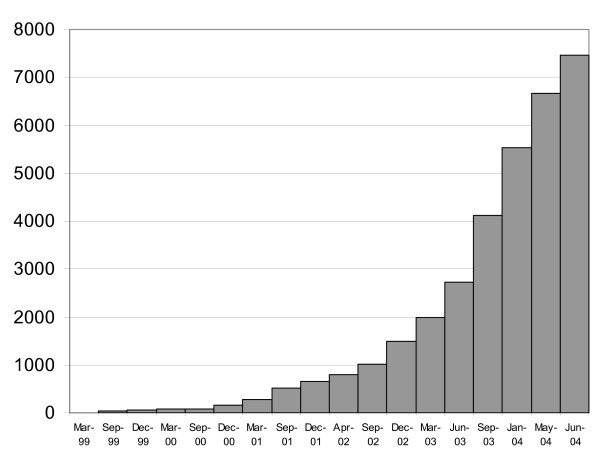
Accrual of cases and controls to GELCAPS.

**Figure 2:**
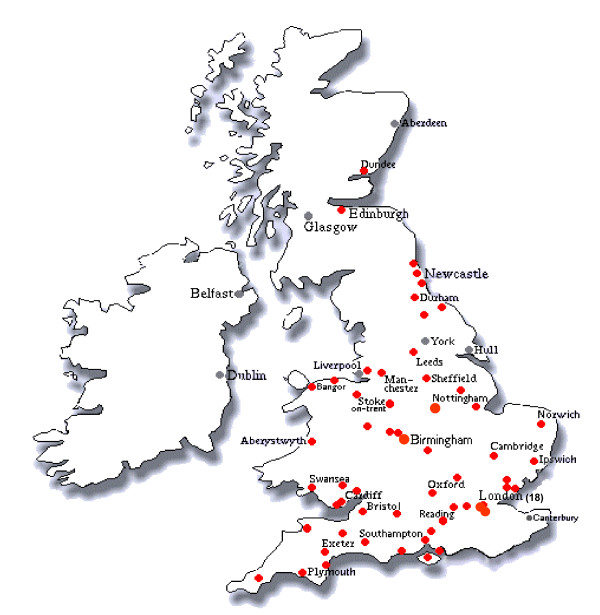
Centres in the UK recruiting to GELCAPS after NCRN adoption.

After securing ethical permissions at a national level through the Multi Research Ethics Committee we started recruitment to GELCAPS in March 1999. Ascertainment of cases was restricted to 28 centres and accrual was maximally 10–20 patients per month. After GELCAPS was incorporated into the NCRN (National Cancer Research Network) portfolio in March 2002 it was subsequently rolled out across England after individual centers had obtained local ethical permissions. Adoption by the NCRN was associated with a significant increase in patient and control accrual (Figure 1). Eventually 140 oncology centers (Figure 2) became active participants in GELCAPS with patient ascertainment averaging ~100 cases per month. The remit and operational procedure by which patients are accrued to NCRN adopted studies does not allow collection of compliance data within each participating center. However, we estimate based on our intimate knowledge of the clinical activities of three centers that patient accrual to GELCAPS is ~70% of those invited to participate.

The original target of GELCAPS was to assemble a series of 2,000 lung cancer cases. Given the efficiency by which samples were being accrued following adoption of GELCAPS by the NCRN a new target of at least 4,000 cases was deemed to be eminently feasible within the time frame for which funding had been secured.

We terminated accrual to GELCAPS in July 2004 by which time samples from 5,269 cases with primary lung cancer and 2,094 controls had been recruited. The majority of cases were male (64%) reflecting the sex preponderance of disease. Whilst the mean age of controls was comparable to cases (62.9 years, SD = 10.6) not surprisingly 69% were female (Table 1). Similarly, the prevalence of smoking was significantly higher amongst cases compared to controls. A high proportion of the cases ascertained had been diagnosed with lung cancer at a young age (Table 1); specifically 1,617 (~31%) of the cases were aged less than 60 years old at diagnosis, compared with < 10% in the general population. The frequency of the various forms of lung cancer was, however, in keeping with that observed in UK general population – ~23% being affected with SCLC and ~73% with NSCLC (Table 1).

**Table 1:** Characteristics of lung cancer patients recruited to GELCAPS

	**Cases**	**Controls**
Total	5,269	2,094
Gender (Male: Female)	3,382 (64.2%)	645 (30.8%)
Age at diagnosis (years)	887 (35.8%)	446 (69.1%)
<40	53 (1.0%)	64 (3.1%)
40–49	302 (5.7%)	166 (7.9%)
50–59	1,146 (21.7%)	514 (24.5%)
60–69	1,868 (35.4%)	756 (36.1%)
70–79	1,590 (30.1%)	510 (24.4%)
80+	310 (5.9%)	84 (4.0%)
Mean (SD)	65.1 (10.0)	62.9 (10.6)
Ethnicity		
Arabic	3 (0.05%)	1 (0.05%)
Asian	7 (0.13%)	6 (0.29%)
Bangladeshi	1 (0.01%)	0
Black-African	4 (0.08%)	1 (0.05%)
Black-Caribbean	31 (0.59%)	8 (0.38%)
Black-Other	1 (0.01%)	0
Indian	16 (0.30%)	4 (0.19%)
Jewish-Ashkenazi	12 (0.22%)	6 (0.29%)
Pakistani	8 (0.15%)	0
White	5,065 (96.1%)	1,947 (93.0%)
Other/not specified	82 (1.6%)	121 (5.8%)
Reported asbestos exposure	807 (15.2%)	125 (6.0%)
Family history of lung cancer	750 (14.2%)	212 (10.1%)
Smoking habits		
Never-smokers	307 (5.8%)	718 (34%)
All smokers		
Age first started smoking (SD)	16.5 (4.0)	17.9 (4.8%)
Pack years in Smokers (SD)	47.2 (30.6)	30.4 (22.1%)
		
Histology of cancer		
Small cell (SCLC)	1,193 (22.6%)	
Non-small cell (NSCLC)	3,815 (72.4%)	
Squamous	1,905 (49.9%)	
Adenocarcinoma (including variants)	1,110 (29.1%)	
Large cell	10 (0.3%)	
Brochoalveolar	44 (1.2%)	
Adenosquamous	11 (0.3%)	
Neuroendocrine	20 (0.5%)	
NSCLC unspecified	715 (18.7%)	
Sarcoma	5 (0.1%)	
Unclassified primary	256 (4.9%)	
		
Tumour stage at presentation, by histology		
SCLC		
Limited	168 (50.4%)	
Extensive	165 (59.6%)	
NSCLC		
I	151 (14.1%)	
II	140 (13.1%)	
III	457 (42.7%)	
IV	323 (30.2%)	

**Figure 3:**
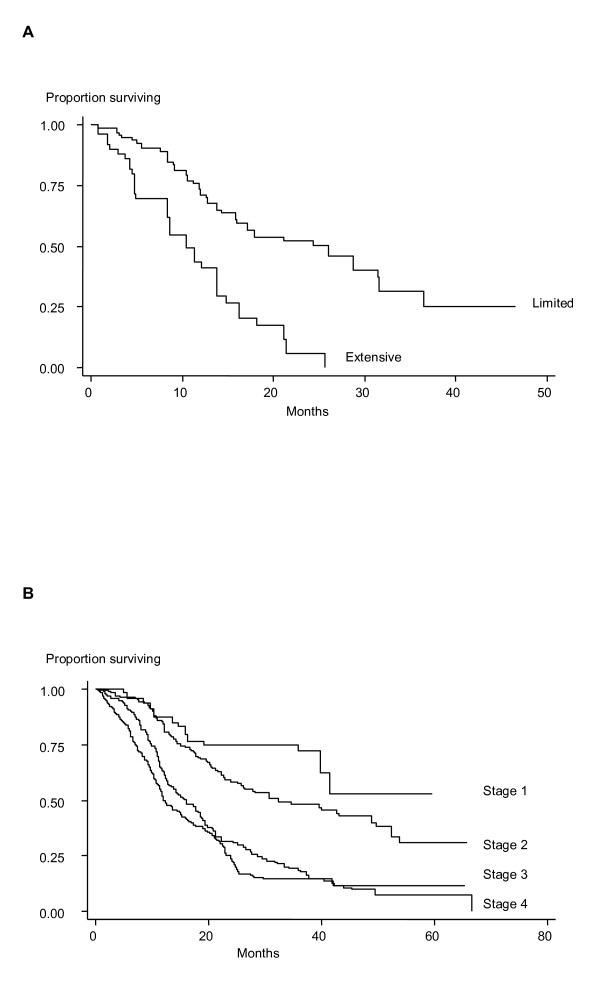
**Survival from lung cancer in patients according to stage at presentation: A) Patients with SCLC, B) Patients with NSCLC**. In both SCLC and NSLC survival was significantly better (P < 0.0001) in patients presenting with early stage disease compared to those presenting with late stage disease in both Log rank tests of the difference in distribution of survival curves and in Cox-proportional hazard test, adjusting for age, sex, year of presentation and treatment with platinum-based chemotherapy. Statistical analyses performed using STATA version 8.0 (College Station, Tx, USA).

To date we have acquired follow up data on 1,187 patients; specifically, information on the staging, management and clinical outcome permitting comparison patients randomly drawn from the general population. Stage at presentation for each of the different subtypes of lung cancer was similar to that observed in the general population; specifically, for patients with SCLC, somewhat less than half (43%) presented with limited disease and of the patients with NSCLC, 13% had stage I, 15% had stage II, 43% had stage III, and 29% had stage IV disease. The majority of patients with limited stage SCLC had been treated with a combination of radical radiotherapy and chemotherapy, whilst all patients received chemotherapy. The main treatment modality for SCLC patients with extensive disease was chemotherapy. Patients with early stage NSCLC (stage I and II disease) were mainly treated with surgical resection of the primary tumor whilst about one third received chemotherapy and radical radiotherapy. The mainstay treatment modality of patients with stage III and IV NSCLC was chemotherapy. Overall the median survival time (MST) for the subset of 1,187 GELCAPS patients was 18.6 months. Prognosis was significantly correlated with stage at presentation, with those presenting early have a far better survival (Figure 3). Patients with SCLC had a MST of 26.0 and 10.5 months if diagnosed with limited and extensive disease respectively. For those with NSCLC, MSTs ranged from 12.1 months for stage IV patients to 32.3 months for stage I disease.

## Discussion

Recent data from GWASs of breast [21,22], prostate [23-27] and colorectal (CRC) cancer [28-31] provides strong evidence for the involvement of common disease-causing alleles and suggests that a relatively large number of genes influence the aetiology in most cancers in the patient population as a whole.

To exploit the advances brought about by the human genome projects, future work in cancer genetics will be dependent upon the acquisition of large well-characterised cohorts of cancer cases. Here we have demonstrated that the centralisation of cancer services in the UK offers an opportunity to establish large, well-characterised cohorts by targeting collection to the largest centres. Moreover mobilising NCRN networks provides a means of delivering consistently the data and sample collection to complete genetic epidemiology studies, relating to the detection of main effects on the required scale.

Because ascertainment of cases through GELCAPS has been based on clinical centres specialising in the treatment of lung cancer a high proportion of cases have been diagnosed young. While this means cases are not fully representative of disease in the general population the distribution of age at diagnosis serves to empower GELCAPS for identifying disease-causing alleles by virtue of genetic enrichment.

Given that constitutional genotypes may well influence patient prognosis it is highly desirable that survivorship is not confounding influence on sample collection. As survival rates in patients recruited to GELCAPS were not significantly different to those documented in previously published audits of lung cancer in the UK there is no evidence that "healthy study participant" selection will have genetically biased ascertainment. For all participants, sex, ethnicity and age at sampling have been documented. The geographical area of birth and area of residence within the UK is known for all of the individuals and this information can be used to allow analyses stratified by region of residence, reducing any effects of population stratification. The possibility of population stratification leading to false inference of disease-genotype association can readily be addressed by adjusting for known region/ethnicity or by using information on unlinked genetic markers.

We acknowledge the potential problem of differential bias in genotyping samples accrued from different sources. Although the samples collected through GELCAPs have been ascertained from many clinical centres we have no evidence that this has affected sample quality as we have previously documented call rates of 99.8% in samples genotyped for 1,500 SNPs [32-35] and Quantile-Quantile plots of test association statistics provide no evidence for differential bias

The NCRN research networks are established within cancer care networks where access to partners is readily available and direct. They are not designed to collect samples from the general population so our choice of collecting samples from partners was a pragmatic one appropriate for the NCRN. Inevitably in studies such as GELCAPS a smaller number of samples from controls will be collected than from cases since in addition to lack of compliance many patients do not have a current partner. The sex of controls ascertained through initiatives such as GELCAPS will usually be of the opposite gender to cases, and controls are potentially over-matched with respect to many lifestyle risk factors. Theses limitations can be offset to a large degree by using samples collected from the healthy spouses/partners of one cancer as a source of controls for a different cancer. This is something we are currently pursuing with respect to a similar NRCN sponsored initiative the National Study of Colorectal Cancer Genetics (NSCCG)

Because of the difficulty of obtaining sufficiently detailed data on environmental exposure in studies such as GELCAPS, and because there are issues to do with comparability of exposure data from controls assembled from different studies, it is acknowledged that studies of environmental risk factors including gene-environment interaction will be limited in resources such as GELCAPS. The main value of collections such as GELCAPS will be in studies of genetic risk factors and gene-gene interactions; hypotheses regarding gene-environment interaction require alternative datasets, such as the European Prospective Investigation into Cancer and nutrition (EPIC) study [36], which are centred around population based-cohorts. Accepting such limitations our experience in developing GELCAPS serves to illustrate how large DNA databases for genetic analyses can rapidly be developed in the UK. At present we have only collected outcome data on around 20% of cases recruited to GELCAPS. By completing the collection of follow up data on all cases we shall be able to assemble a unique series for examining the influence of constitutional genotype on clinical outcome in the population setting.

## Conclusion

Finally, it is noteworthy that the value of GELCAPS has been demonstrated in a recent GWAS of lung cancer we have conducted in which we have been able to robustly identify a susceptibility variant for the disease mapping to 15q [37].

## Abbreviations

GELCAPS: Genetic Lung Cancer Predisposition Study

## Compteting interests

The authors declare that they have no competing interests.

## Authors' contributions

TE and RSH were the principal investigators for the GELCAPS, devised the study. AM helped in study development and was responsible for database design, and management of study coordinators. All authors contributed to the paper.

## Availability & requirements

Cancer Research UK: 

GELCAPS: 

National Cancer Research Network: 

NHS Cancer Plan: 

National Study of Colorectal Cancer Genetics: 

European Prospective Investigation into Cancer and Nutrition: 

## Pre-publication history

The pre-publication history for this paper can be accessed here:


